# *Bacillus siamensis* CCT8089: a novel phosphate-solubilizing bacterium enhancing maize and soybean growth

**DOI:** 10.3389/fpls.2025.1671339

**Published:** 2025-10-16

**Authors:** Mirela Mosela, Galdino Andrade, Alison Fernando Nogueira, Lycio Shinji Watanabe, Silas Mian, Matheus Felipe de Lima Andreata, Marcos Ventura Faria, Liliane Scislowski, Daniel Fernando Viana Fagundes, Antony Wallace Marcos, Henry Boguschi Cava, Pablo Diego Silva Cabral, Roger Wisniewski Da Conceição, Sérgio Vicente de Azevedo, Liliam Silvia Candido, Leandro Afonso, Rafael de Assis, Leandro Simões Azeredo Gonçalves

**Affiliations:** ^1^ Microbiology Department, Universidade Estadual de Londrina (UEL), Londrina, Paraná, Brazil; ^2^ Agronomy Department, Universidade Estadual de Londrina (UEL), Londrina, Paraná, Brazil; ^3^ Chemical Departament, Universidade Estadual de Londrina (UEL), Londrina, Paraná, Brazil; ^4^ Agronomy Department, Universidade Estadual do Centro Oeste (UNICENTRO), Guarapuava, Paraná, Brazil; ^5^ Agronomy Department, Instituto Federal Goiano (IFG), Rio Verde, Goiás, Brazil; ^6^ Biology Department, Instituto Federal de São Paulo (IFSP), Barretos, São Paulo, Brazil; ^7^ Biology Department, Universidade Federal de Grande Dourados (UFGD), Dourados, Mato Grosso do Sul, Brazil; ^8^ Structural Biology Department, Universidade Estadual de Ponta Grossa (UEPG), Ponta Grossa, Paraná, Brazil

**Keywords:** bioinoculants, organic acids, phosphate-solubilizing bacteria (PSB), phosphorus solubilization, seed- and in-furrow inoculation, yield stability

## Abstract

Phosphorus (P) is an essential macronutrient for plant growth, but its availability in tropical soils is limited due to fixation by iron and aluminum oxides. This study aimed to characterize the *Bacillus siamensis* CCT8089 strain *in vitro*, evaluating its phosphate-solubilizing and plant growth-promoting abilities and its agronomic potential in maize and soybean crops. *In vitro*, CCT8089 tolerated osmotic stress, produced exopolysaccharides and biofilm, and demonstrated compatibility with commercial inoculants (*Azospirillum brasilense* and *Bradyrhizobium japonicum*). The strain also exhibited biocontrol activity against *Macrophomina phaseolina* and *Sclerotinia sclerotiorum*. CCT8089 produced organic acids, particularly malic and acetic acid in Ca_3_(PO_4_)_2_, while gluconic acid and lactic acid were predominant in FePO_4_ and AlPO_4_, respectively. Additionally, CCT8089 produced indole-3-acetic acid (IAA) and significantly increased root biomass in greenhouse experiments. In field trials, CCT8089 applied via seed treatment or furrow application significantly increased yield and phosphorus acquisition efficiency in maize and soybean compared to the non-inoculated control (83.25 kg P_2_O₅). Furrow application was more effective for maize, while no significant differences were observed between application methods in soybean. Notably, CCT8089 demonstrated greater yield stability across different environmental conditions, with the best performance observed at 200 mL/ha in-furrow application and 100 mL per 60,000 seeds (maize) or 50 kg of seeds (soybean) for seed treatment. These results highlight the potential of strain CCT8089 as a novel inoculant for maize and soybean crops, with viable application through both seed treatment and furrow application.

## 1 Introduction

Phosphorus (P) is an essential macronutrient for plant growth and development, integral to various important biomolecules, such as DNA, RNA, ATP, NADPH, and phospholipids. Additionally, P is directly involved in respiration and photosynthesis processes (Lincoln et al., 2023). The predominant form of P uptake by roots is orthophosphate (Pi, H_2_PO_4_⁻/HPO_4_²⁻), which is translocated to the aerial parts via the xylem. In highly weathered soils, including most Brazilian soils, less than 3% of the total P is readily available to plants (Pavinato et al., 2020). This low availability is mainly attributed to the fixation of inorganic P by the reactive surfaces of Fe and Al oxides, which predominate in tropical soils (Rodrigues et al., 2016; Pavinato et al., 2020). In acidic environments, such as tropical soils, P strongly binds to these metals, reducing their mobility and making them unavailable to plants (Haynes, 1982; Tiessen, 2015).

The application of phosphate fertilizers in tropical regions is essential to meet the nutritional demands of crops. However, this practice is inefficient, as only ~10-30% of the applied P is absorbed by crops. At the same time, a large portion remains in the soil, forming a substantial reservoir of residual P, known as legacy P, which accumulates in forms that are not readily available to plants (Rodrigues et al., 2016; Pavinato et al., 2020; Gotz et al., 2024). In Brazilian soils, it is estimated that approximately 33.4 Tg (teragrams; 1Tg = 10^12^ g = 1 Mt) of P accumulated between 1967 and 2016, and this amount could reach up to 106.5 Tg by 2050 if fertilizer use continues at the current rate (Pavinato et al., 2020). Furthermore, global phosphorus reserves are limited and concentrated in a few regions, making tropical agriculture highly dependent on imports and susceptible to geopolitical fluctuations and dollar volatility (Mosela et al., 2022). For example, in Brazil, approximately 72% of the phosphate fertilizer used is imported, reflecting a high dependence on external sources to meet national demand (Brasil, 2022).

Several approaches have been developed to enhance phosphorus use efficiency (PUE) in agriculture. Among the adopted strategies, the development of cultivars with greater phosphorus use efficiency stands out, as these plants demonstrate a higher capacity for P uptake and utilization (Lambers, 2022; Pavinato et al., 2024). Precision agriculture techniques, which adjust the dosage, placement, and timing of fertilizer application, are also crucial for optimizing P absorption and minimizing losses due to fixation and leaching (Bindraban et al., 2020; Silva et al., 2024). Additionally, practices that promote soil health, such as the addition of organic matter and the use of microbial inoculants that solubilize phosphorus, have shown a positive effect in increasing P availability (Mosela et al., 2022; Raniro et al., 2023).

Phosphate-solubilizing microorganisms (PSMs) play a crucial role in the phosphorus cycle in soil by converting insoluble phosphorus forms, such as Ca, Fe, and Al phosphates, as well as organic phosphorus compounds, into orthophosphates, thereby increasing PUE in crops (Rawat et al., 2021; Sindhu et al., 2022). The main mechanisms used by these microorganisms to solubilize and mineralize phosphorus include the production and excretion of organic acids and the action of hydrolytic enzymes such as phytases and phosphatases. Organic acids, such as citric, oxalic, and malic acids, are excreted by microorganisms and acidify the rhizosphere environment, promoting phosphorus release through proton substitution and metal ion complexation (Panchal et al., 2021; Pang et al., 2024). Phytases and phosphatases, in turn, are essential for mineralizing organic phosphorus in soils. Phytase catalyzes the degradation of phytic acid into free phosphate and less phosphorylated myo-inositols. Similarly, acidic and alkaline phosphatases complement this process by hydrolyzing other organic phosphate compounds, maximizing PUE (Rizwanuddin et al., 2023; Vashishth et al., 2023).

Several species of bacteria, actinobacteria, and fungi have been reported for their ability to solubilize phosphate, contributing to increased nutrient availability for plants. Among fungi, the genera *Aspergillus, Penicillium, Trichoderma* and *Talaromyces* stand out as important phosphate solubilizers, while for bacteria, the key genera include *Bacillus, Pseudomonas, Acinetobacter, Streptomyces, Burkholderia* and *Paraburkholderia* (Sarmah and Sarma, 2023). Many commercial products based on phosphate-solubilizing microorganisms have been registered in Brazil, with *Bacillus* and *Pseudomonas* being the main genera employed (Andreata et al., 2024). *Bacillus* strains are widely recognized for their properties as plant growth-promoting rhizobacteria (PGPR), acting not only in phosphate solubilization and mineralization but also in the production of bioactive metabolites such as siderophores and phytohormones. Additionally, members of this genus are excellent root colonizers, capable of surviving under various stress conditions and exhibiting biocontrol activities against a wide range of soilborne phytopathogens (Saxena et al., 2020; Mosela et al., 2022; de Oliveira-Paiva et al., 2024; Mian et al., 2024; Andreata et al., 2025).

The present study aimed to characterize the *Bacillus siamensis* CCT8089 strain *in vitro*, exploring its growth-promoting properties and organic acid production, and to evaluate its agronomic potential as a novel phosphate-solubilizing agent and growth promoter in maize and soybean crops. Additionally, the study investigated the efficacy of this strain under different application methods, seed treatment and in-furrow application, aiming to optimize its performance in agricultural systems.

## 2 Material and methods

### 2.1 Bacterial strain

The *Bacillus siamensis* CCT8089 strain, part of the microorganism collection of the company BIOINPUT, is deposited at the Fundação André Tosello – Tropical Culture Collection (https://fat.org.br). Isolated from maize rhizospheric soil, this strain was selected due to its remarkable phosphate-solubilizing ability and antifungal activity against soilborne phytopathogenic fungi, including *Rhizoctonia solani, Macrophomina phaseolina*, and *Fusarium solani*. The *Bacillus megaterium* CNPMS B119 (B119) and *B. subtilis* CNPMS B2084 (B2084) strains, used as controls, are part of the multifunctional microorganisms and phytopathogens collection (CMMF) of Embrapa Maize and Sorghum (de Oliveira-Paiva et al., 2024) and are components of the BiomaPhos product, developed by Embrapa in partnership with the company Bioma (Paraná, Brazil).

### 2.2 *In vitro* analysis

#### 2.2.1 Osmotic stress tolerance

To evaluate osmotic stress tolerance, the strains were inoculated on plates containing Tryptone Soy Agar (TSA) medium at 10% (w/v), supplemented with 405 g/L of sorbitol, and incubated at 30°C for 72 h (Vieira Velloso et al., 2020). This medium has a reduced water activity (0.919 Aw), allowing the selection of bacteria capable of growing under osmotic stress conditions.

#### 2.2.2 Exopolysaccharide production

Exopolysaccharide (EPS) production was determined using 5 µL of standardized bacterial suspensions (0.5 on the McFarland scale), applied onto sterile filter paper discs (5 mm in diameter) placed on plates containing LBA medium. The plates were incubated overnight at 28°C, and the formation of mucoid colonies around the discs was observed. The mucus was scraped off and transferred to tubes containing absolute ethanol (2 mL) to confirm EPS production. A precipitate indicated EPS production, while turbidity in the solution was considered a negative result (Paulo et al., 2012).

#### 2.2.3 Biofilm formation

The biofilm formation capacity was evaluated according to the method described by Stepanović et al. (2007) with adaptations. Bacterial cultures were incubated in BHI broth for 24 hours at 37°C with agitation at 100 rpm. Then, 200 µL of these suspensions were added in triplicate to 96-well plates. The B119 and B2084 strains were positive controls, while the medium without bacterial inoculum was a negative control. After subsequent incubation under the same conditions, the bacterial suspensions were removed, and the wells were washed three times with 250 µL of sterile saline solution (0.85% NaCl). For fixation, 200 µL of methanol was added to the wells for 15 minutes, followed by removing the content and drying the plates for 30 minutes at 54°C. Subsequently, 200 µL of crystal violet was added for 5 minutes. The excess dye was removed, and the wells were washed until no residual staining was visible. For quantification, 200 µL of ethanol was added to the wells, and 100 µL of the resulting solution was transferred to a new plate. Absorbance was measured at 570 nm using an ELISA reader. The optical densities of the isolates (ODi) were compared to the optical density of the negative control (ODc), allowing the classification of the isolates as biofilm producers.

#### 2.2.4 Siderophore production

Bacterial strains were evaluated for their siderophore production capacity using the universal CAS assay (Schwyn and Neilands, 1987), with modifications developed by Hu (2011). Bacterial cultures were grown in 10% TSB at 28°C with shaking at 150 rpm for 72 and 120 hours. After incubation, the fermented cultures were centrifuged at 12,000 rpm for 10 minutes, with the pellet discarded and the cell-free supernatant (CFS) used for siderophore estimation. Next, 0.5 mL of each CFS was mixed with 0.5 mL of CAS reagent. After 20 minutes of reaction, optical density was measured at 630 nm, and the produced siderophore was quantified as a percentage of the siderophore unit (PSU) (Arora and Verma, 2017).

#### 2.2.5 Indole-3-acetic acid production

To quantify the production of indole-3-acetic acid (IAA), the strains were cultured in a Tryptone Soy Broth (TSB) medium supplemented with 1.0 mg/mL of tryptophan. The cultures were incubated at 30°C, 100 rpm, in the dark for 5 days. After incubation, the cultures were centrifuged for 10 minutes at 5,500 rpm, and 1 mL of the supernatant was mixed with 1 mL of Salkowski reagent (de Sousa et al., 2021). The mixture was incubated at 30°C in the dark for 20 min to develop the characteristic pink-to-magenta coloration of the IAA–Salkowski complex (FeCl_3_ in 35% HClO_4_); uninoculated blanks remained pale yellow/orange. The IAA concentration was then determined by measuring absorbance at 540 nm against an IAA standard curve.

#### 2.2.6 Intra- and extracellular phytase production

The intra- and extracellular phytase production analysis was performed according to the protocol described by Bhandari et al. (2023), with modifications. Bacterial strains were cultured in NBRIP medium (National Botanical Research Institute’s Phosphate Growth Medium) and incubated on an orbital shaker at 30°C and 150 rpm for 96 hours. After incubation, cultures were centrifuged at 5,000 rpm and 4°C for 30 minutes. The supernatant was used to assess extracellular phytase activity, while the cell pellet was processed for intracellular phytase analysis. The cell pellet was resuspended in 2 mL of 50 mmol/L acetate buffer (pH 5) containing 2 mmol/L CaCl_2_, frozen at –80°C for 30 minutes, and subsequently thawed in a water bath at 42°C for 15 minutes. The resulting cell suspension was sonicated for 3 minutes and then centrifuged at 10,000 rpm and 4°C for 30 minutes. The supernatant obtained from cell lysis was collected and refrigerated for intracellular phytase determination.

To determine intra- and extracellular phytase activity, 100 µL of 0.2 mol/L acetate buffer (pH 4.5) and 80 µL of 0.25 mol/L sodium phytate solution were added to 15 mL tubes and vortexed for 10 seconds. Then, 200 µL of the sample was added and incubated at 37°C for 20 minutes. After incubation, 800 µL of 10% (v/v) trichloroacetic acid (TCA) and 900 µL of the AAM reagent mixture (1 mol/L ascorbic acid, 0.6 mol/L H_2_SO_4_, and 0.5% ammonium molybdate in a 1:1:1 v/v/v ratio) were added. Samples were homogenized and incubated at 37°C for 20 minutes, protected from light. All analyses were performed in triplicate for each sample, using acid-washed glassware, with measurements also conducted in triplicate and results expressed as the mean of three readings. Absorbance was measured using a spectrophotometer at 820 nm, and phytase activity was expressed in mU/mL.

#### 2.2.7 Phytate mineralization

Phytate mineralization was assessed using the same extracellular phytase setup, except that strains were grown in the phytase-quantification medium supplemented with inositol hexaphosphate (phytic acid). In a Falcon tube, 200 µL of each sample was added to 800 µL of 10% TCA, followed by homogenization. Subsequently, 900 µL of the AAM solution was added, the mixture was homogenized again, and samples were incubated at 37°C for 20 minutes. All assays were performed in triplicate for each sample, with triplicate measurements and results expressed as the mean of three readings. Absorbance was measured at 820 nm using a spectrophotometer, and phytate mineralization was expressed in mg/L.

#### 2.2.8 Acid and alkaline phosphate production

Acid and alkaline phosphatase activity was determined according to the method described by Tabatabai and Bremner (1969). The respective culture media were included for background measurements, avoiding potential interference in constructing calibration curves. Bacterial strains were grown in NBRIP medium supplemented with three different phosphate sources (NBRIP + Ca_3_(PO_4_)_2_, NBRIP + FePO_4_, or NBRIP + AlPO_4_), and cultures were then centrifuged at 4,500 rpm for 10 minutes. Next, 1.0 mL of the resulting supernatant or control solution was transferred to Falcon tubes. Then, 4.0 mL of the appropriate buffer solution was added: 0.1 mol/L acetate buffer (pH 5) for acid phosphatase or 0.1 mol/L Na_2_CO_3_/NaHCO_3_ buffer (pH 10) for alkaline phosphatase. The mixture was homogenized by vortexing, and 0.29 mL of 0.05 mol/L p-nitrophenyl phosphate (pNPP) substrate solution was added. Samples were incubated at 37°C for 1 hour. After incubation, 1.0 mL of 0.5 mol/L CaCl_2_ and 4 mL of 0.5 mol/L NaOH were added, followed by vortexing and centrifugation at 4,500 rpm for 10 minutes. All assays were performed in triplicate for each sample, with measurements also conducted in triplicate, and results were expressed as the mean of the three readings. Absorbance was measured at 400 nm using a spectrophotometer, and phosphatase activity was expressed in µg pNPP/mL/h.

#### 2.2.9 Compatibility test between bacterial strains

In the modified cross-streak assay, strains were streaked on YMA (for *Bradyrhizobium japonicum* SEMIA 5079) or RC (for *Azospirillum brasilense* Ab-V5/Ab-V6) plates and incubated at 28°C for 24–96h (until confluent growth). Incompatibility was scored by the presence of an inhibition zone at the intersection of the streaks.

#### 2.2.10 Direct antagonism by dual culture

To evaluate the antagonistic potential of the bacterial strains, two phytopathogenic fungi, *Macrophomina phaseolina* and *Sclerotinia sclerotiorum*, were used. The fungi were pre-activated on Potato Dextrose Agar (PDA) medium and incubated in a growth chamber (B.O.D.) at 25°C with a 12h light/12h dark photoperiod for 72 hours. PDA plates were marked with two equidistant points, 1 cm from the edge for the antagonism assay. The bacterial strains were inoculated at these points using a bacteriological loop. In contrast, the fungi were inoculated by placing a mycelial disc between the bacterial inoculation points in the center of the plate. The plates were then incubated again at 25°C with a 12h light/12h dark photoperiod for 3 to 5 days, depending on the growth rate of each pathogen. Plates containing only the fungus served as controls. A growth inhibition zone around the bacterial strains was used as a criterion to determine antagonism.

### 2.3 Organic acid production

The determination of organic acids was adapted from the method described by Yan et al. (2013). Bacterial strains were cultured in NBRIP medium supplemented with three different phosphate sources (NBRIP + Ca_3_(PO_4_)_2_, NBRIP + FePO_4_, or NBRIP + AlPO_4_). Aliquots of the liquid culture media were collected and analyzed for organic acid content using high-performance liquid chromatography (HPLC) on a Shimadzu LC-20A system (Kyoto, Japan). The system consisted of a high-pressure pump (LC-20AT), an autosampler (SIL-20AC HT), a refractive index detector (RID-10A), a photodiode array detector (SPD-M20A), a column oven (CTO-20A), and a system controller module (CBM-20A). Chromatographic separation was performed using a Phenomenex 5 µm C18 MG column (250 × 4.6 mm) (Phenomenex, Inc., Torrance, CA, USA). The mobile phase was a 25 mM sodium phosphate buffer adjusted to pH 2.4, and elution was carried out isocratically at a flow rate of 1.0 mL/min. The column temperature was maintained at 40°C, and the injection volume was 20 µL. Detection was performed simultaneously using the refractive index detector (RID-10A) and the photodiode array detector (SPD-M20A), at a fixed wavelength of 205 nm and a spectral scan from 200 to 400 nm. Data acquisition and processing were carried out using Shimadzu’s LC solution software (Kyoto, Japan). Organic acid standards included oxalic, gluconic, malic, lactic, acetic, citric, and succinic acids.

### 2.4 Scanning electron microscopy

To analyze the colonization capacity of strain CCT8089, soybean seeds were surface disinfected by immersion in 1.5% NaOCl for 10 minutes, followed by five washes with sterile deionized water. The seeds were treated with 200 mL of CCT8089 cell culture fermentate per 100 kg of seeds. As a negative control, surface-disinfected but untreated seeds were used. After treatment, the seeds were incubated for 7 days on moistened germination paper with sterilized distilled water and maintained in a growth chamber at 25°C and 70% relative humidity. After this period, five germinated seeds from each treatment were selected for sample preparation, focusing on the root area. The samples were fixed with 2.5% (v/v) glutaraldehyde in 0.10 M sodium cacodylate buffer for 4 hours, followed by three washes with distilled water. Then, the samples were dehydrated in acetone solutions of increasing concentrations (30, 50, 70, 90, and 100% v/v) for 10 minutes at each concentration, with the final dehydration step performed in duplicate. After dehydration, the samples were dried in a CO_2_ critical point dryer (BALTEC CPD 030), mounted on metal stubs, and gold-sputter-coated (BALTEC SDC 050) for imaging by scanning electron microscopy (SEM; Shimadzu SUPERSCAN SS-550, Japan).

### 2.5 Greenhouse experiment

The CCT8089 strain, stored at −80°C in cryotubes containing liquid TSB and glycerol at a 2:1 ratio, was reactivated on Petri plates containing LBA culture medium at 30°C for 24 hours. A pre-inoculum was prepared from pure colonies suspended in saline solution (0.85% sodium chloride). The turbidity was adjusted to 0.5 on the McFarland nephelometric scale (1.5 × 10⁸ CFU mL⁻¹). Thirty microliters of these bacterial suspensions were transferred to 125 mL Erlenmeyer flasks containing 30 mL of Ag-03 culture medium (g/L: Yeast Extract 10; Tryptone 5; Glucose 20; CaCl_2_ 0.147; FeSO_4_ 0.2; MgCl_2_ 0.5; K_2_HPO_4_ 1.5; MnSO_4_ 0.2; NaCl 0.2; pH 7.0), with 5% inoculum, incubated for 72 hours at 37°C with shaking at 200 rpm. After fermentation, the product concentration was adjusted to 1.0 × 10^9^ CFU mL⁻¹.

The seeds of the maize cultivar P3340VYHR (Corteva^®^) were inoculated with 100 mL per 60,000 seeds. The seeds were then sown in 10-L tapered polyethylene pots (height 25 cm; top inner diameter 27 cm; bottom inner diameter 20 cm), each with 8 drainage holes (8–10 mm Ø) and no saucer, filled with a 1:1 (v/v) washed sand:soil mixture, and the experiment was conducted in the greenhouse at the Universidade Estadual de Londrina (UEL). The experimental design was completely randomized, with ten replications. Treatments, both inoculated and non-inoculated with strain CCT8089, were irrigated every two days, and plants were collected 45 days after sowing. The evaluated characteristics included shoot and root dry mass.

### 2.6 Field experiment

In the field trials, Pioneer P3310VYHR maize and DM66I68IPRO soybean seeds were used to evaluate the efficacy of two biological products, *Bacillus velezensis* CCT8089 and BiomaPhos*^®^*, applied using two methods: seed treatment and furrow application. For seed treatment, the tested doses were as follows: i) BiomaPhos (100 mL per 60,000 maize seeds and 100 mL per 50 kg of soybean seeds), and CCT8089 (50 mL, and 100 mL per 60,000 maize seeds, and 50 mL, and 100 mL per 50 kg of soybean seeds). For furrow application, the tested doses were: i) BiomaPhos (200 mL/ha for both crops), and CCT8089 (100 mL and 200 mL/ha for both crops). The spray volume used for furrow applications was 50 L/ha. In all experiments, biological phosphate-solubilizing products were evaluated only at 83.25 kg P_2_O₅ ha⁻¹. As controls (no biological product), two treatments with phosphate fertilization only were included at 83.25 and 111 kg P_2_O₅ ha⁻¹. In all soybean experiments, *Bradyrhizobium japonicum* strains SEMIA 5079 and 5080 were inoculated at a dose of 100 mL per 50 kg of seeds.

For the maize experiments, five trials were conducted: 1) Londrina – PR (2023/2024 season), 2) Guarapuava – PR (2023/2024 season), 3) Rio Verde – GO (2024/2024 season), 4) Barretos – SP (2024/2024 season), and 5) Dourados – MS (2024/2024 season). For soybean, five trials were conducted: 1) Faxinal – PR, 2) Londrina – PR, 3) Guarapuava – PR, 4) Rio Verde – GO, and 5) Barretos – SP. All soybean experiments were conducted during the 2023/2024 season. **Supplementary Table S1** presents the physicochemical soil analyses and other characteristics related to the evaluation sites.

The experiment was conducted in a randomized complete block design, with four replications. Each plot consisted of eight rows, each seven meters long, spaced 0.45 m apart. Before the experiment was established, the maize and soybean trial areas were fertilized with 100 kg KCl ha⁻¹ and 20 kg N ha⁻¹. Topdressing fertilization was carried out for maize with 138 kg N ha⁻¹ at the V6 developmental stage.

Five representative plants from each experimental plot were collected at the physiological maturity stage. To determine the phosphorus (P) content in grains (maize and soybean) and the shoot biomass (maize), the samples were dried in an oven at 70°C for 72 hours and ground using a Willey MA340 knife mill (Piracicaba, São Paulo, Brazil).

Subsequently, 0.1 g aliquots were digested in a nitroperchloric acid solution, following the method described by Malavolta et al. (1989). The P content was determined using the molybdenum blue spectrophotometric method (Pradhan and Pokhrel, 2013), with readings performed on an Agilent 8453 spectrophotometer (Agilent Technologies, California, USA) at a wavelength of 660 nm.

Grain yield (kg ha⁻¹) was determined after the plants were harvested from the six central rows of each plot. For maize, the components of phosphorus use efficiency (PUE) were determined according to Moll et al. (1982), while for soybeans, the phosphorus content in the grains was measured. Phosphorus uptake efficiency (PUpE, in g of P absorbed per g of P applied) was calculated as the ratio between the total plant P and the available P for the plant. Phosphorus utilization efficiency (PUtE, in g of grain produced per g of total P in the plant) was determined as the ratio between the dry biomass of the grains and the total amount of P in the plant. PUE (in g of grain produced per g of P applied) was calculated as the product of PUpE and PUtE.

### 2.7 Data analysis

Agronomic data were subjected to analysis of variance (ANOVA), and if the assumptions were met, means were compared using Tukey’s test. For yield data, the stability index proposed by Lin and Binns (1988) was calculated:


$$P_{i\_a}=\left[\frac{\sum_{j=1}^{n}{\left(Y_{ij-}Y_{gi}\right)}^{2}}{2n}\right]\frac{CV_{j}}{CV_{T}}$$

$$P_{i\_f}=\left[\frac{\sum_{j=1}^{f}{\left(Y_{ij-}Y_{gi}\right)}^{2}}{2f}\right]\frac{CV_{j}}{CV_{T}}$$

$$P_{i\_u}=\left[\frac{\sum_{j=1}^{f}{\left(Y_{ij-}Y_{gi}\right)}^{2}}{2u}\right]\frac{CV_{j}}{CV_{T}}$$

where: Pi__a_ is the stability statistic defined by Lin and Binns (1988), Pi_f and Pi_u are statistics defined by Cruz and Carneiro (Cruz and Carneiro, 2003), f and u represent the number of favorable (positive environmental index, including zero, as defined by Eberhart and Russell (1966)) and unfavorable (negative environmental index) environments, respectively, with n = f + u, Y_ij_ is the phenotypic value of genotype *i* in environment *j*, Yg_i_ is the ideal response of a hypothetical genotype in environment *j*, estimated using the bi-segmented model of Cruz et al. (1989), CV_j_ and CV_T_ correspond to the residual coefficient of variation for environment *j* and the sum of the coefficients of variation for all environments, respectively. All analyses were performed using R software, utilizing the Metan package (Olivoto and Lúcio, 2020) and the AgroR package (Shimizu et al., 2025).

## 3 Results

### 3.1 *In vitro* analysis

**Figure 1:**
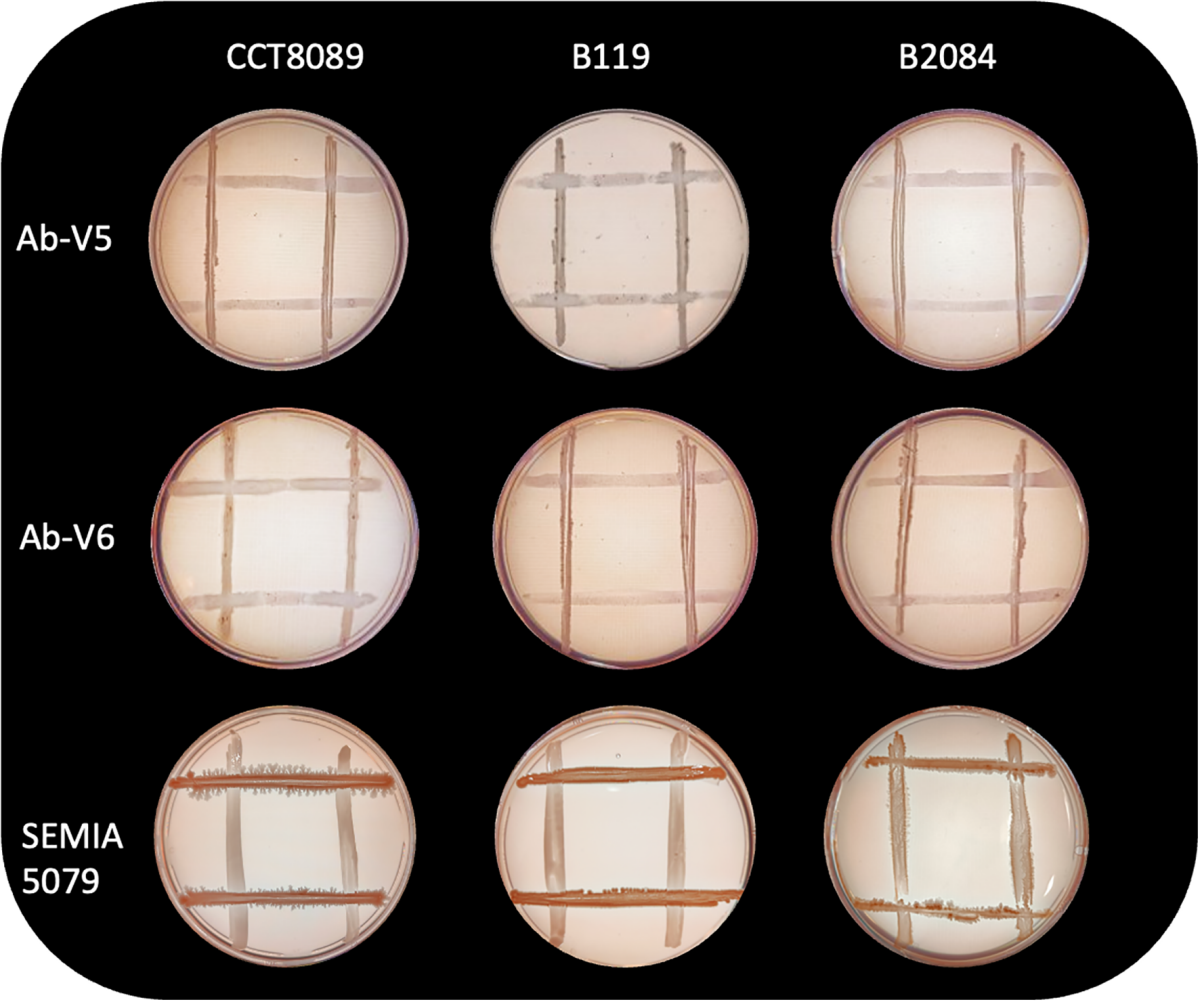
Compatibility between *Bacillus* strains (*B. siamensis* CCT8089, *B*. *subtilis* B119, and *B*. *megaterium* B2084) with *Azospirillum brasilense* (Ab-V5 and Ab-V6) and *Bradyrhizobium japonicum* (SEMIA 5079).

**Figure 2:**
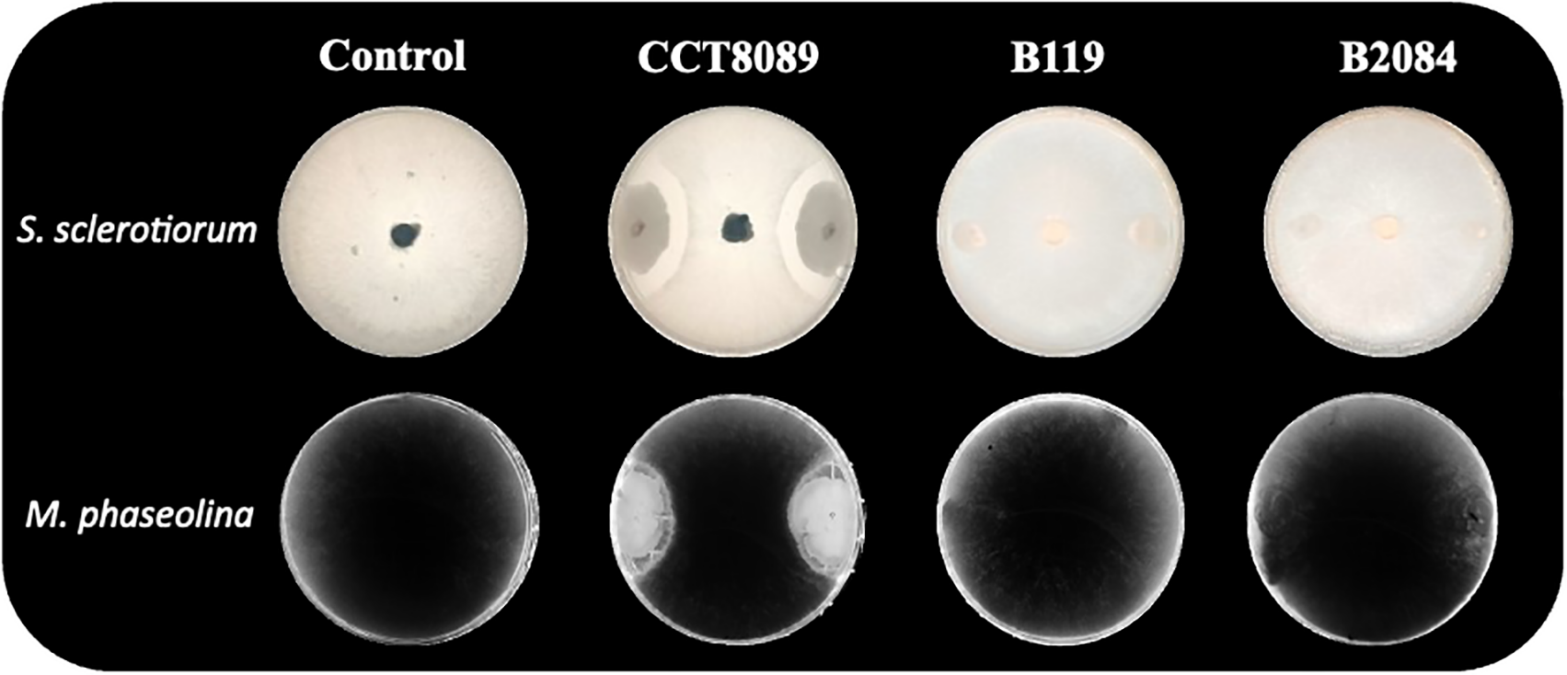
Direct antagonism in dual culture of *Bacillus* strains (*B. siamensis* CCT8089, *B*. *subtilis* B119, and *B*. *megaterium* B2084) and the fungi *Sclerotinia sclerotiorum* and *Macrophomina phaseolina*.

The *in vitro* analysis showed that all three *Bacillus* strains (CCT8089, B119, and B2084) demonstrated the ability to tolerate water stress, as well as produce exopolysaccharides (EPS) and biofilm (**Table 1**). Additionally, these strains were compatible with *Azospirillum brasilense* (Ab-V5 and Ab-V6) and *Bradyrhizobium japonicum* (SEMIA 5079) (**Figure 1**). However, only strain CCT8089 exhibited antagonistic activity against the phytopathogenic fungi *Sclerotinia sclerotiorum* and *Macrophomina phaseolina* (**Figure 2**).

**Table 1:** Growth promotion characteristics and antagonism against phytopathogenic fungi of *Bacillus siamensis* CCT8089, *B. megaterium* B119, and *B. subtilis* B2084.

Characteristics	*B. siamensis* CCT8089	*B. subtilis* B119	*B. megaterium* B2084
Water Stress Tolerance	+	+	+
Exopolysaccharide (EPS) Production	+	+	+
Biofilm Production	+	+	+
Siderophore Production (24h) (psu)	7.66	21.77	19.22
Siderophore Production (72h) (psu)	18.11	31.33	31.33
IAA Production (µg mL^-1^)	8.08	4.73	46.32
Extracellular Phytase (mU mL^-1^)	8.31	3.09	2.85
Intracellular Phytase (mU mL^-1^)	49.12	37.39	34.18
Phytate mineralization (mg L^-1^)	23.17	17.59	24.12
Acid Phosphatase (μg pNP mL^-1^ h^-1^) - Ca_3_(PO_4_)_2_	1160.00	1220.00	1230.00
Acid Phosphatase (μg pNP mL^-1^ h^-1^) – FePO_4_	1436.16	1274.66	1344.41
Acid Phosphatase (μg pNP mL^-1^ h^-1^) – AlPO_4_	1440.00	990.00	1130.00
Alkaline Phosphatase (μg pNP mL^-1^ h^-1^) - Ca_3_(PO_4_)_2_	1100.00	1350.00	1190.00
Alkaline Phosphatase (μg pNP mL^-1^ h^-1^) – FePO_4_	1350.02	958.63	1117.18
Alkaline Phosphatase (μg pNP mL^-1^ h^-1^) – AlPO_4_	1120.00	930.00	963.45
Combatibility (Ab-V5 and Ab-V6)	+	+	+
Combatibility (SEMIA 5079)	+	+	+
Antagonism against *Rhizoctonia solani*	+	–	–
Antagonism against *Macrophomina phaseolina*	+	–	–

(+) and (-) indicate positive and negative results for the evaluated characteristics, respectively.

For siderophore production, strains B119 and B2084 exhibited the highest values.

Regarding indole-3-acetic acid (IAA) production, the highest value was observed for strain B2084 (46.32 µg mL⁻¹), followed by strains CCT8089 and B119 (8.08 and 4.73 µg mL⁻¹, respectively). Strains CCT8089, B119, and B2084 exhibited extracellular phytase activities of 8.31, 3.09 and 2.85 mU mL⁻¹, respectively, while intracellular phytase activities were 49.12, 37.39 and 34.18 mU mL⁻¹, respectively. For phytate mineralization, these strains showed values of 23.17, 17.50, and 24.12 mg L⁻¹, respectively.

All strains showed activity for both acid and alkaline phosphatases. For acid phosphatase, the highest activity values were observed with FePO_4_ and AlPO_4_ sources, especially for strain CCT8089, with 1436.16 and 1440.00 µg pNP mL⁻¹ h⁻¹, respectively. For Ca_3_(PO_4_)_2_, the highest values were observed for strains B2084 and B119. Regarding alkaline phosphatase, strain CCT8089 also showed the highest activity with FePO_4_ and AlPO_4_ as phosphorus sources, while for Ca_3_(PO_4_)_2_, the highest activity was observed for strain B119.

**Figure 3:**
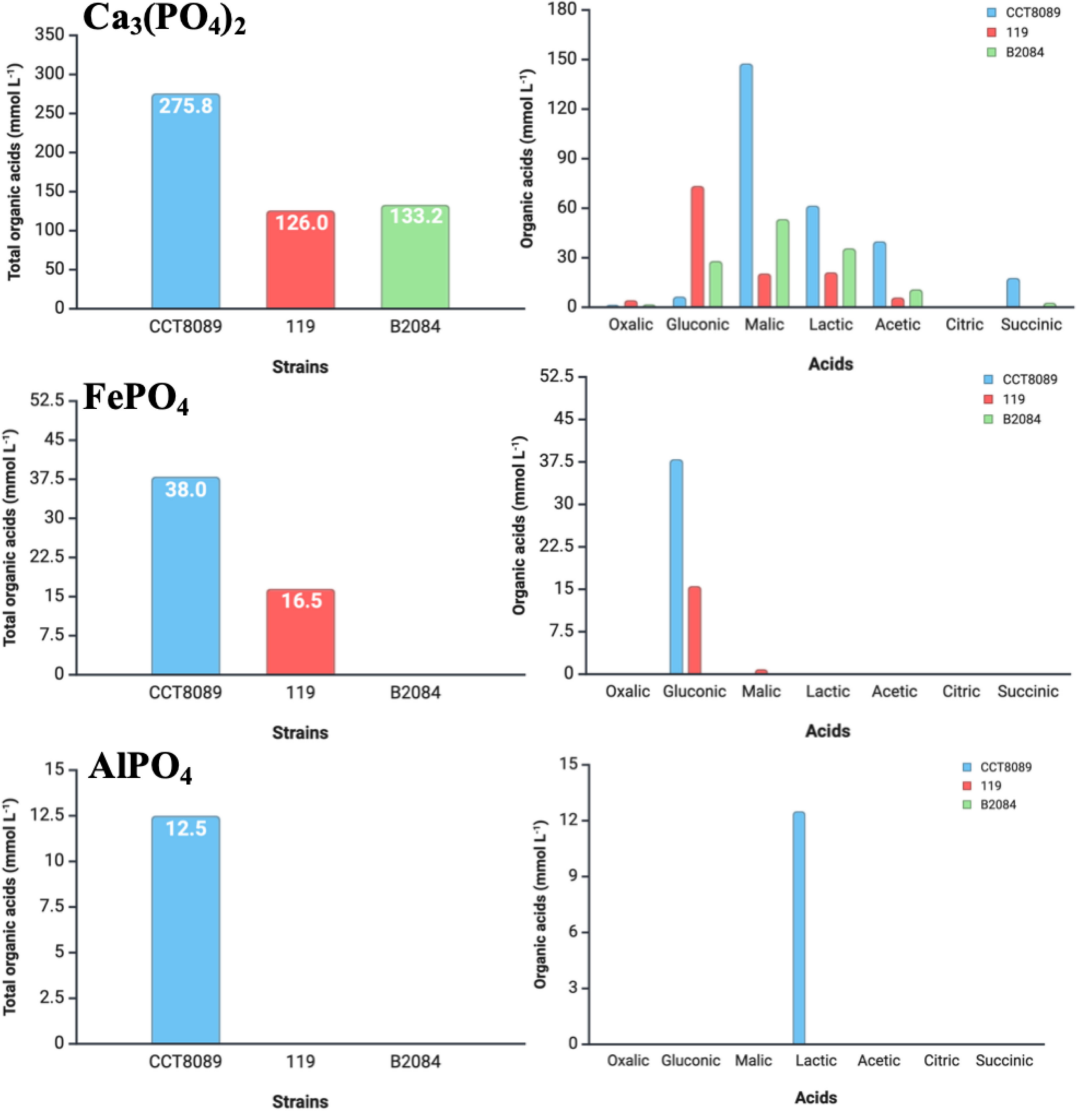
Organic acid production (mmol L⁻¹) in the fermentates of *Bacillus siamensis* CCT8089, *B*. *subtilis* B119, and *B*. *megaterium* B2084 in NBRIP (National Botanical Research Institute’s Phosphate) medium with Ca_3_(PO_4_)_2_, FePO_4_, and AlPO_4_.

For organic acid production, strains CCT8089, B119, and B2084 exhibited a higher capacity to produce organic acids when using Ca_3_(PO_4_)_2_ as the phosphate source compared to FePO_4_ and AlPO_4_ (**Figure 3**). Strain CCT8089 demonstrated the highest organic acid production capacity, with 275.8, 38.0, and 12.5 mmol L⁻¹ in Ca_3_(PO_4_)_2_, FePO_4_, and AlPO_4_, respectively, followed by strain B119, which produced 126.0 and 16.5 mmoL L⁻¹ in Ca_3_(PO_4_)_2_ and FePO_4_, respectively. Strain B2084 produced 171.0 mmoL L⁻¹ in Ca_3_(PO_4_)_2_, while its organic acid production in FePO_4_ and AlPO_4_ was not detectable.

For Ca_3_(PO_4_)_2_, strain CCT8089 exhibited the highest production of malic acid (147.7 mmoL L⁻¹), followed by lactic, acetic, succinic, gluconic, and oxalic acids (61.7, 40.1, 18.0, 6.7, and 1.6 mmoL L⁻¹, respectively). In strain B119, the predominant organic acid produced was gluconic acid (73.53 mmoL L⁻¹), followed by lactic, malic, acetic, and oxalic acids (21.3, 20.6, 6.1, and 4.4 mmoL L⁻¹, respectively). Meanwhile, strain B2084 mostly produced malic acid (53.5 mmoL L⁻¹), followed by lactic, gluconic, acetic, succinic, and oxalic acids (35.8, 28.2, 11.0, 2.9, and 1.9 mmoL L⁻¹, respectively). For FePO_4_, strains CCT8089 and B119 predominantly produced gluconic acid (38.0 and 15.6 mmoL L⁻¹, respectively), whereas for AlPO_4_, strain CCT8089 mainly produced lactic acid (12.5 mmoL L⁻¹).

### 3.2 Root colonization in *in vivo* assays

**Figure 4:**
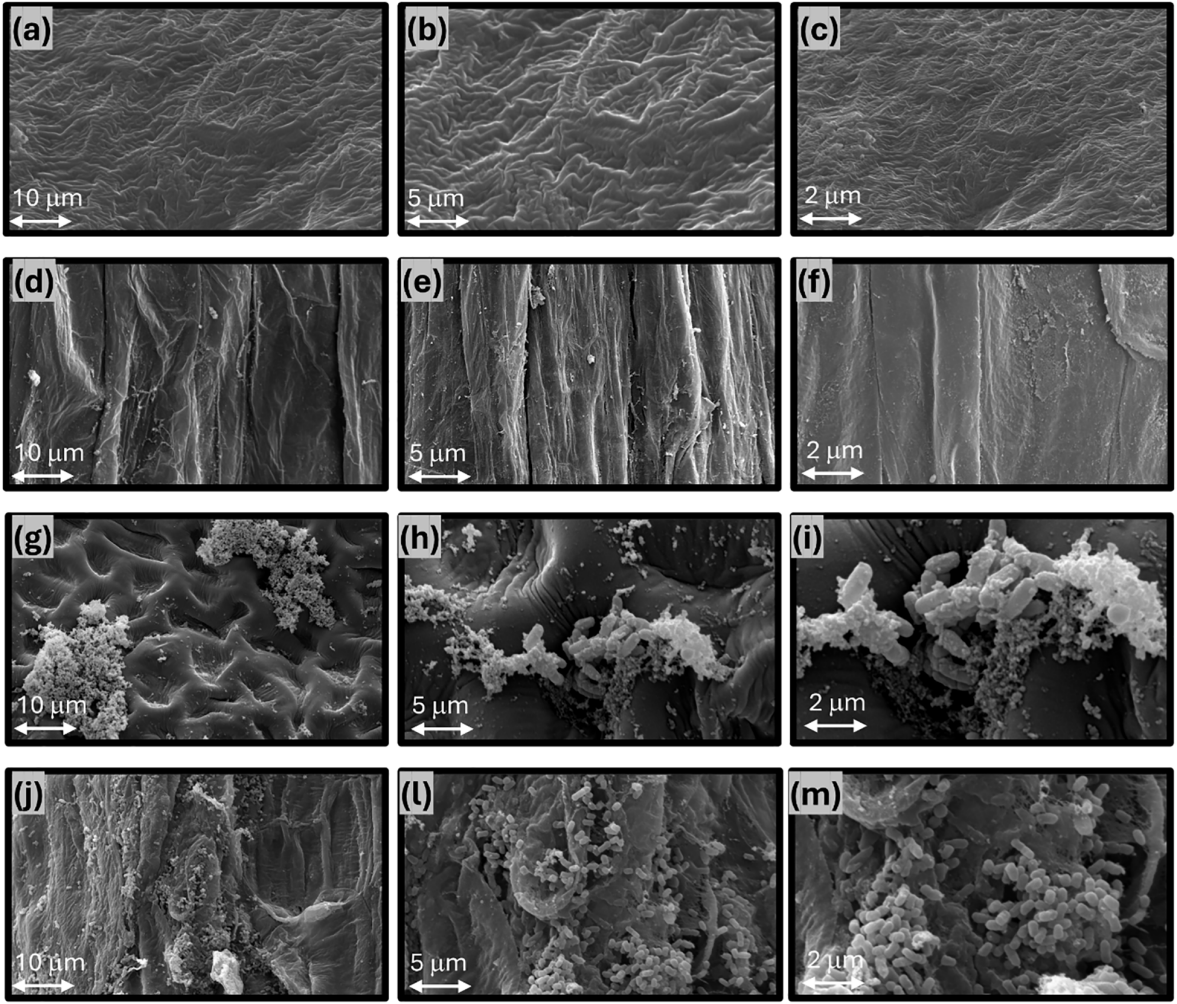
Scanning electron microscopy of soybean seed surfaces and root colonization with and without treatment by *Bacillus siamensis* CCT8089, with magnifications of 1000x, 3000x, and 7000x, respectively. Panels **(a–c)** represent the untreated seed, while panels **(d–f)** show the corresponding untreated roots after seven days of germination. Panels **(g–i)** display seeds treated with CCT8089 (200 mL/100 kg of seeds) at a concentration of approximately 1 × 10^9^ CFU mL⁻¹, and panels **(j–m)** show the colonized roots after seven days.

Scanning electron microscopy images revealed no microorganisms on non-inoculated seed coats or roots (**Figures 4a–f**). In contrast, seeds treated with *Bacillus siamensis* CCT8089 showed abundant rod-shaped cells adhered along seed-coat ridges and depressions, forming microcolonies (**Figures 4g–i**). Seven days after sowing, inoculated seedlings exhibited extensive colonization of the root epidermis and root hairs (**Figures 4j–m**). Cells were frequently observed embedded in an amorphous extracellular material and arranged in clusters bridging surface features, consistent with early biofilm formation.

### 3.3 Greenhouse experiment

**Figure 5:**
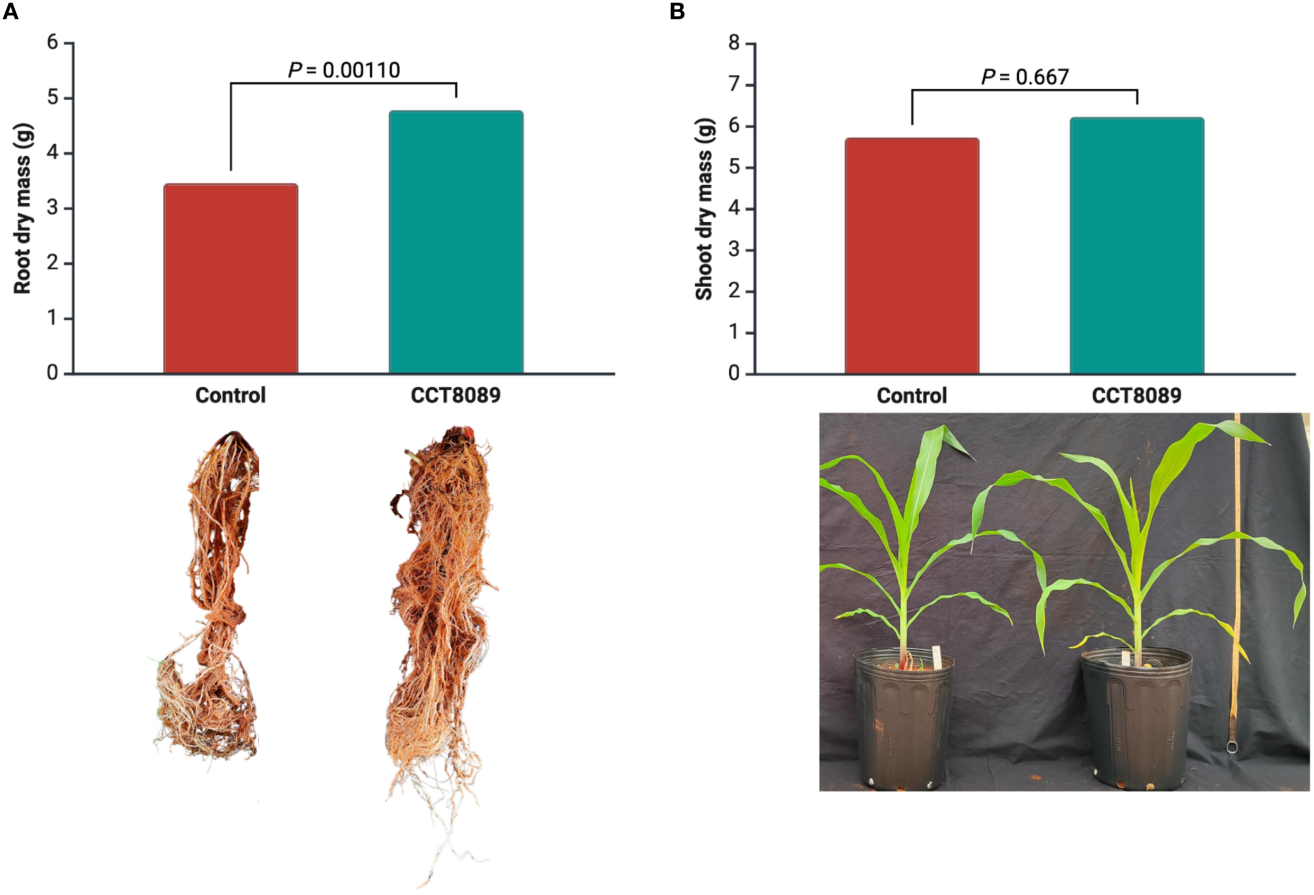
Effect of *Bacillus siamensis* CCT8089 on maize shoot and root dry biomass. **(A)** Shoot and root dry mass of inoculated and non-inoculated maize plants. **(B)** Representative images of the root system and plant growth.

In the greenhouse experiment, strain CCT8089 significantly increased the root dry mass of maize plants compared to the control treatments (**Figure 5**). Plants inoculated with CCT8089 showed a 38.3% increase in root biomass compared to the non-inoculated control. For shoot dry mass, no significant differences were observed between treatments.

### 3.4 Field experiment – yield

For the maize field experiments, a significant effect was observed for all sources of variation (**Table 2**). The coefficient of variation was 15%, indicating good experimental precision. Regarding average yield, most treatments inoculated with *Bacillus* strains showed higher productivity compared to the non-inoculated control (83.25 kg of P_2_O₅). Furrow application (FA) was 9.11% more effective than seed treatment (SD). Among the SD treatments, strain CCT8089 (50 and 100 mL/100 kg of seeds) and BiomaPhos (100 mL/100 kg of seeds) resulted in yield increases of 8.2%, 8.5%, and 1.5%, respectively, compared to the control (83.25 kg of P_2_O₅). For the FA treatments, CCT8089 (100 and 200 mL/ha) and BiomaPhos (200 mL/ha) led to yield increases of 10.19%, 22.82%, and 14.18%, respectively, compared to the control (83.25 kg of P_2_O₅).

**Table 2:** Analysis of variance for yield in maize and soybean experiments with inoculation of phosphate-solubilizing bacteria via seed treatment and furrow application.

Source of variation	Maize		Soybean	
	DF	Mean Square	DF	Mean Square
Repetitions/Environment	15	1.04 x10^6^	15	1.6x10^5^
Treatments (T)	7	3.21x10^6^ (p<0.001)	7	5.7x10^5^ (p<0.001)
Environment (E)	4	2.97x10^8^ (p<0.001)	4	4.0x10^7^ (p<0.001)
T x E	28	2.35x10^6^ (p<0.001)	28	3.2x10^5^ (p<0.001)
Error	105	7.58x10^8^	105	1.1x10^5^
CV (%)	15.00		9.48	
Mean				
Control (111 Kg de P_2_O_5_)		5684	3539	
Control (83.25 Kg de P_2_O_5_)		5360	3319	
BiomaPhos SD (100mL)		5439 (1.47%)	3644 (9.80%)	
CCT8089 SD (50 mL)		5815 (8.49%)	3338 (0.57%)	
CCT8089 SD (100 mL)		5801 (8.23%)	3656 (10.15%)	
BiomaPhos FA (200mL)		6120 (14.18%)	3441 (3.68%)	
CCT8089 FA (100 mL)		5906 (10.19%)	3573 (7.65%)	
CCT8089 FA (200 mL)		6583 (22.82%)	3815 (14.94%)	

^1/^the percentual value represents the comparison of treatments relative to the control treatment (83.25 kg of P_2_O₅).

When analyzing the results by location, the CCT8089 FA (200 mL) treatment showed the highest average yield in the environments of Londrina, Guarapuava, Rio Verde, and Barretos (**Table 3**). In Londrina, in addition to CCT8089 FA (200 mL), the treatments BiomaPhos (SD and FA) and CCT8089 FA (100 mL) also stood out in terms of yield increase. In Guarapuava, the best results were observed for the control (111 kg of P_2_O₅), BiomaPhos FA, and CCT8089 FA (100 mL) treatments. In the Rio Verde and Dourados environments, most treatments with biological products led to increased yield compared to the control (83.25 kg of P_2_O₅), except for BiomaPhos SD, which did not perform well. In Barretos, besides CCT8089 FA (200 mL) being a standout, the Control (111 kg of P_2_O₅) and CCT8089 SD (100 mL) treatments also performed well.

**Table 3:** Mean comparison test for yield and stability analysis according to Lin and Binns in maize experiments with inoculation of phosphate-solubilizing bacteria applied via seed treatment and furrow application in different environments.

Treatments	Yield (Kg/ha)^1/^					Lin e Binns’ superiority index		
	Londrina (23/24)	Guarapuava (23/24)	Rio Verde (24/24)	Barretos (24/24)	Dourados (24/24)	Pi_a	Pi_f	Pi_u
Control (111 Kg de P2O5)	7537.3 c	10079.9 ab	4488.6 bc	3709.1 a	2604.078 bc	801963 (4)	981075 (4)	682555 (6)
Control (83.25 Kg de P2O5)	8158.3 bc	8848.6 c	4239.9 c	3475.4 b	2011.994 c	1370791 (7)	1729107 (7)	1131914 (8)
BiomaPhos SD (100mL)	8600.9 ab	7580.1 c	4435.9 bc	3474.6 b	2525.795 bc	1526183 (8)	2669078 (8)	764254 (7)
BiomaPhos FA (200mL)	8845.6 ab	11400.2 a	4888.0 abc	2730.7 c	2736.26 b	393277 (2)	7108 (2)	650723 (5)
CCT8089 SD (50 mL)	8059.3 bc	8939.3 bc	5185.2 abc	3175.4 b	3716.895 a	842159 (5)	1741980 (5)	242278 (3)
CCT8089 SD (100 mL)	7490.6 c	8908.2 bc	5865.3 ab	3721.3 a	3017.188 b	913653 (6)	2132882 (6)	100834 (1)
CCT8089 FA (100 mL)	8459.9 ab	10267.5 ab	5889.3 ab	2332.2 c	2581.994 bc	531961 (3)	397515 (3)	621593 (4)
CCT8089 FA (200 mL)	9014.2 a	11270.9 a	6171.2 a	3869.1 a	2589.001 bc	128886 (1)	4178 (1)	212024 (2)

^1^Means followed by the same letter in the column do not differ statistically according to Tukey’s test (*p* < 0.10).

²*Pi_ₐ*, Superiority index for a broad environment; *Pi_f*, Superiority index for favorable environments; *Pi_u*, Superiority index for unfavorable environments. ³Numbers in parentheses indicate the ranking of treatments in terms of stability under environmental variations.

Most treatments with biological products demonstrated greater yield stability, both in general environments and under favorable and unfavorable conditions, compared to the control treatment (83.25 kg of P_2_O₅). Under general and favorable environments conditions, the treatments CCT8089 FA (200 mL), BiomaPhos FA (200 mL), and CCT8089 FA (100 mL) stood out with the highest stability indices. Conversely, in unfavorable environments, the treatments CCT8089 SD (100 mL), CCT8089 FA (200 mL), and CCT8089 SD (50 mL) showed the best performance in terms of yield stability.

For soybean cultivation, a significant effect was observed for all sources of variation, with a low coefficient of variation (**Table 2**). Overall, treatments with biological inoculants yielded higher yields than the non-inoculated control (83.25 kg of P_2_O₅). However, no significant differences were detected between the application methods of the biological products. In the SD application treatments, CCT8089 (100 mL) and BiomaPhos stood out, with yield increases of 10.1% and 9.8%, respectively, compared to the control. For the FA application, CCT8089 (200 mL) exhibited the highest yield increase, with a 14.9% improvement.

In Faxinal, most treatments with biological products resulted in yield increases compared to the non-inoculated control (83.25 kg of P_2_O₅), except for the CCT8089 SD (50 mL) treatment (**Table 4**). In Londrina, only the CCT8089 FA (200 mL) treatment showed a significant increase compared to the control. In Guarapuava, the best-performing treatments were the control (111 kg of P_2_O₅), BiomaPhos SD (100 mL), BiomaPhos FA (200 mL), and CCT8089 SD (100 mL). The highest yield increases were observed in the Rio Verde and Barretos environments with BiomaPhos SD (100 mL) and CCT8089 FA (100 and 200 mL). In Rio Verde, the CCT8089 SD (50 and 100 mL) treatments also exhibited superior performance compared to the control.

**Table 4:** Mean comparison test for yield and stability analysis according to Lin and Binns in soybean experiments with inoculation of phosphate-solubilizing bacteria applied via seed treatment and furrow application in different environments.

Treatments	Yield (Kg/ha)^1/^					Lin e Binns’ superiority index ^2,3/^		
	Faxinal (23/24)	Londrina (23/24)	Guarapuava (23/24)	Rio Verde (23/24)	Barretos (23/24)	Pi_a	Pi_f	Pi_u
Control (111 Kg de P2O5)	5386.1 a	2517.1 bc	4222.3 a	3281.8 bc	2286.7 cd	112139 (5)	3529 (2)	184546 (6)
Control (83.25 Kg de P2O5)	4426.8 b	2569.2 bc	3931.9 b-d	3311.2 bc	2356.1 cd	217297 (8)	311611 (8)	154420 (4)
BiomaPhos SD (100mL)	5504.9 a	2322.3 c	4098.6 ab	3437.1 ab	2858.3 ab	94612 (4)	3822 (3)	155139 (5)
BiomaPhos FA (200mL)	5470.9 a	2748.2 b	4080.2 a-c	3053.5 c	1854.1 e	179749 (6)	5333 (4)	296027 (8)
CCT8089 SD (50 mL)	4622.2 b	2521.9 bc	4045.3 a-d	3423.6 ab	2076.5 de	215730 (7)	202587 (7)	224491 (7)
CCT8089 SD (100 mL)	5435.9 a	2582.9 bc	4135.2 ab	3615.1 a	2508.8 bc	67074 (2)	3084 (1)	109735 (2)
CCT8089 FA (100 mL)	5301.2 a	2467.8 bc	3845.0 d	3484.4 ab	2765.3 ab	87145 (3)	45962 (6)	114600 (3)
CCT8089 FA (200 mL)	5483.8 a	3266.5 a	3862.0 cd	3515.6 ab	2945.9 a	14016 (1)	32562 (5)	1652 (1)

^1^Means followed by the same letter in the column do not differ statistically according to Tukey’s test (*p* < 0.10).

²*Pi_ₐ*, Superiority index for a broad environment; *Pi_f*, Superiority index for favorable environments; *Pi_u*, Superiority index for unfavorable environments.

³Numbers in parentheses indicate the ranking of treatments in terms of stability under environmental variations.

In soybean cultivation, greater yield stability was also observed with the use of biological products compared to the non-inoculated control (83.25 kg of P_2_O₅), except in unfavorable environments. In general environments, the treatments with the highest stability were CCT8089 FA (200 mL), CCT8089 SD (100 mL), and CCT8089 FA (200 mL). In favorable environments, the treatments that stood out for their stability were CCT8089 SD (100 mL), Control (111 kg of P_2_O₅), and BiomaPhos SD (100 mL). On the other hand, in unfavorable environments, the highest stability was observed in CCT8089 FA (200 mL), CCT8089 SD (100 mL), and CCT8089 FA (100 mL) treatments.

### 3.5 Field experiment – phosphorus use efficiency

**Figure 6:**
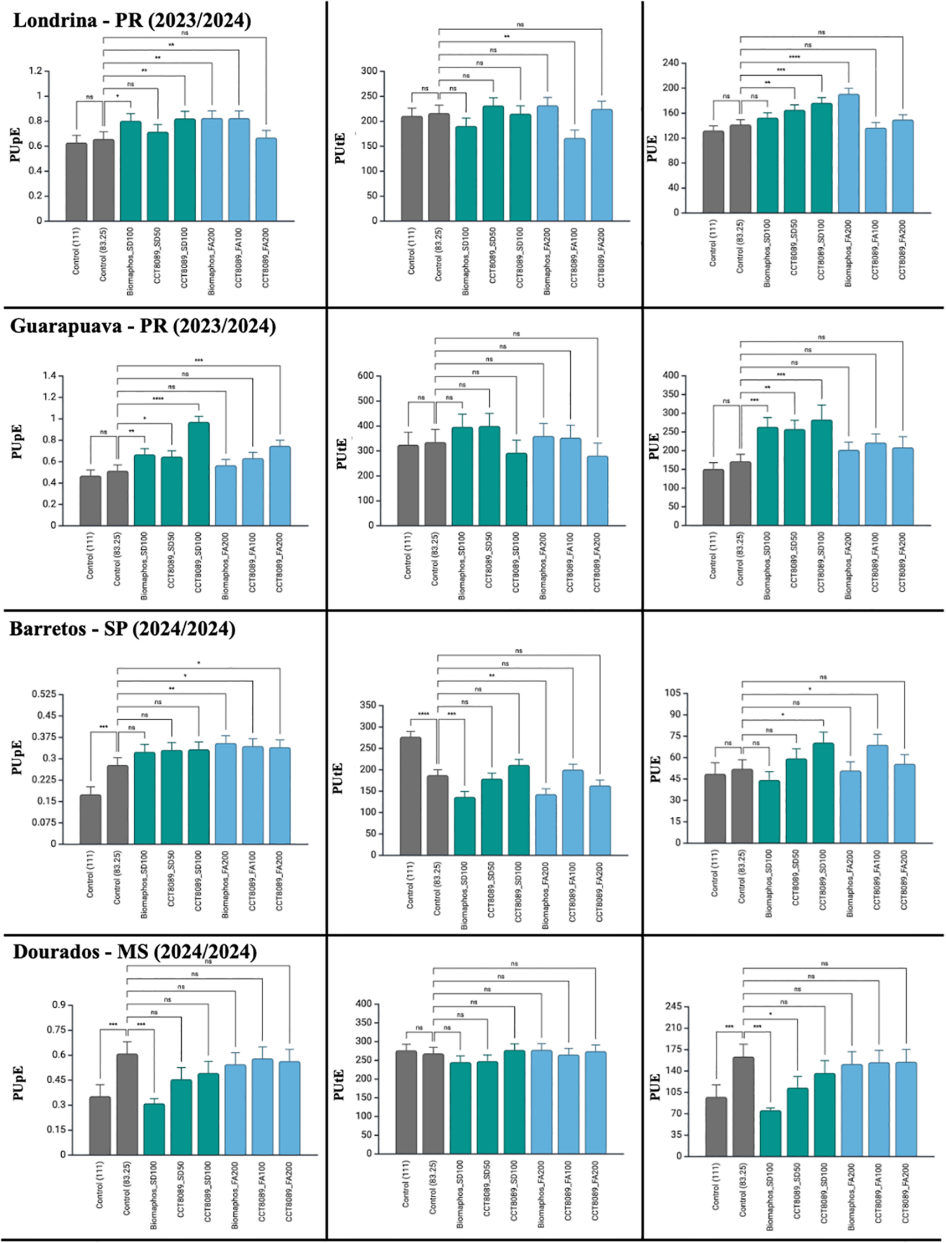
Tukey’s mean comparison test for phosphorus acquisition efficiency (PUpE), phosphorus utilization efficiency (PUtE), and phosphorus use efficiency (PUE) in maize experiments inoculated with phosphate-solubilizing bacteria in different environments. Significance (Tukey HSD, adjusted p-values): ns = p ≥ 0.05; ^*^p< 0.05; ^**^p< 0.01; ^***^p< 0.001; ****p < 0.0001. Asterisks above a bar indicate the treatment differs from its fertilizer-only control at the same P_2_O₅ rate within the same environment.

In the maize experiments, all sources of variation had a significant effect on PUpE and PUE (**Table 5**). However, for phosphorus utilization efficiency (PUtE), no significant differences were detected between treatments. The coefficients of variation were 18.55, 19.37 and 23.2% for PUpE, PUtE, and PUE, respectively. Most treatments with biological inoculants showed higher PUpE and PUE values compared to the non-inoculated control (83.25 kg of P_2_O₅). No significant differences were observed between the application methods of the biological products in the field for these traits. In SD treatments, CCT8089 (100 mL) exhibited the highest efficiency indices for both PUpE and PUE. For FA application, CCT8089 (100 mL) stood out for PUpE, while BiomaPhos (200 mL) showed the best performance for PUE. When analyzed by environment, a variation in response was observed for PUpE and PUE, indicating that environmental conditions influenced these traits (**Figure 6**).

**Table 5:** Analysis of variance for phosphorus acquisition efficiency (PUpE), utilization efficiency (PUtE), and use efficiency (PUE) in maize and soybean experiments inoculated with phosphate-solubilizing bacteria in different environments.

Source of Variation	DF	Maize (Mean Square)			Soybean (Mean Square)
		PUpE	PUtE	PUE	PUE
Repetitions/ Environment	12	0.023	6431	2110	23.3
Treatments (T)	7	0.084 (p<0.001)	2217 (p=0.48)	4183 (p<0.001)	366.1 (p<0.001)
Environment (E)	3	1.159 (p<0.001)	152816 (p<0.001)	144057 (p<0.001)	5412.5 (p<0.001)
T x E	21	0.033 (p<0.001)	5359 (p=0.005)	3020 (p<0.001)	44.9 (p<0.001)
Error	84	0.010	2382	1060	16.0
CV (%)		18.55	19.37	23.2	9.71
Mean^1/^					
Control (111 Kg de P_2_O_5_)		0.41	272.31	107.45	31.88
Control (83.25 Kg de P_2_O_5_)		0.51	252.00	132.38	39.87
BiomaPhos SD (100mL)		0.53 (2.33%)	242.31	134.24 (1.40%)	43.78 (9.81%)
CCT8089 SD (50 mL)		0.54 (4.46%)	264.81	148.86 (12.44%)	40.09 (0.56%)
CCT8089 SD (100 mL)		0.66(27.18%)	249.31	166.75 (25.96%)	43.91 (10.13%)
BiomaPhos FA (200mL)		0.57(11.26%)	253.25	148.99 (12.55%)	41.34 (3.70%)
CCT8089 FA (100 mL)		0.60(15.73%)	246.44	145.51 (9.92%)	42.91 (7.62%)
CCT8089 FA (200 mL)		0.58(12.62%)	235.94	142.30 (7.49%)	45.82 (14.92%)

^1/^the percentual value represents the comparison of treatments relative to the control treatment (83.25 kg of P_2_O₅).

**Figure 7:**
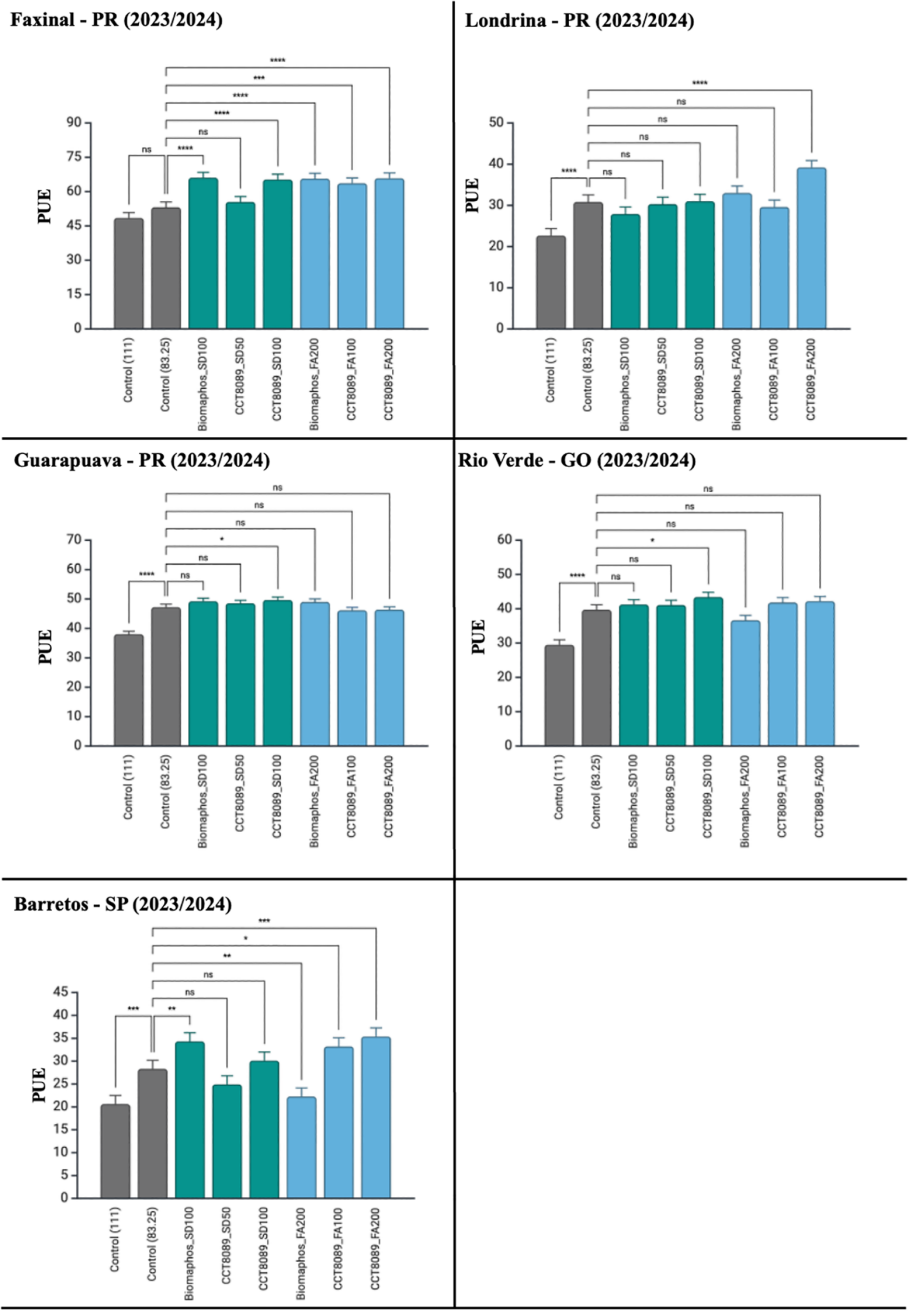
Tukey’s mean comparison test for phosphorus use efficiency (PUE) in soybean experiments inoculated with phosphate-solubilizing bacteria in different environments. Significance (Tukey HSD, adjusted p-values): ns = p ≥ 0.05; ^*^p< 0.05; ^**^p< 0.01; ^***^p< 0.001; ****p < 0.0001. Asterisks above a bar indicate the treatment differs from its fertilizer-only control at the same P_2_O₅ rate within the same environment.

A significant effect was observed in soybean cultivation for all sources of variation related to PUE (**Table 5**). Most treatments with biological inoculants showed higher PUE values than the non-inoculated control (83.25 kg of P_2_O₅), with no differences between application methods. For seed treatment (SD), the highest PUE values were recorded for CCT8089 SD (100 mL) and BiomaPhos SD (100 mL). For furrow application (FA), CCT8089 (200 mL) was the most efficient treatment. When analyzed by the environment, CCT8089 FA (200 mL) showed a significant difference from the control in Faxinal, Londrina, and Barretos (**Figure 7**). Similarly, CCT8089 SD (100 mL) demonstrated a significant difference from the control in Faxinal, Guarapuava, and Rio Verde.

## 4 Discussion

*Bacillus* spp. are widely recognized for its role in nutrient solubilization and plant growth promotion, making it a key genus for the development of agricultural inoculants (Tiwari et al., 2019; Poveda and González-Andrés, 2021; Etesami et al., 2023; Patani et al., 2024). In this study, we evaluated the potential of *Bacillus siamensis* CCT8089 as a promising agent for phosphate solubilization and plant growth promotion in maize and soybean crops. Based on *in vitro* results, CCT8089 demonstrated tolerance to water stress and the ability to produce EPS and biofilm, key traits for adaptation and colonization of the rhizosphere environment (Beauregard et al., 2013; Knights et al., 2021). This colonization ability was confirmed through SEM images, where cellular aggregates were observed in soybean root tissues, highlighting the strain’s capacity to establish itself in the plant rhizosphere.

The CCT8089 strain demonstrated compatibility with other well-established commercial inoculants in Brazil, such as *A. brasilense* (Santos et al., 2021) and *B. japonicum* (Zeffa et al., 2020). This compatibility is important as it allows for the synergistic interaction between these microorganisms, enhancing their benefits to the host plant (Bargaz et al., 2021; Wang et al., 2023). Additionally, the strain exhibited antagonistic activity against important soilborne phytopathogenic fungi (*R. solani* and *M. phaseolina*), highlighting its multifunctional potential.

The ability of the three *Bacillus* strains (CCT8089, B119, and B2084) to produce phytase and phosphatase highlights their potential to hydrolize organic phosphorus compounds, making phosphate more available for plant uptake. Phytate, one of the primary forms of organic phosphorus in the soil, can account for up to 50% of total organic phosphorus (Vashishth et al., 2023). In this context, the presence of phytase-producing bacteria, such as certain *Bacillus* strains, can enhance phosphorus availability by releasing inorganic P from phytate compounds (Ramesh et al., 2011; Vashishth et al., 2023; Shao et al., 2025). In addition to phytase, phosphatase (acidic and alkaline) also plays a crucial role in phosphorus mobilization, as it catalyzes the hydrolysis of phosphomonoester bonds, converting organic P into plant-available forms (Singh and Satyanarayana, 2011). Ramesh et al. (2011) demonstrated that inoculating soybean seeds with different *Bacillus* isolates increased phosphatase activity in the rhizospheric soil, and this increase was positively correlated with higher phosphorus levels in the plant.

The greater organic-acid production by strains CCT8089 and B119 with Ca_3_(PO_4_)_2_, relative to FePO_4_ and AlPO_4_, likely reflects differences in P-source solubility coupled with strain-level metabolic regulation (Rawat et al., 2021; de Oliveira-Paiva et al., 2024; Massucato et al., 2025). Fe- and Al-phosphates are typically mobilized only at lower pH and/or in the presence of strong metal-chelating acids, which imposes higher metabolic demand on microorganisms (proton extrusion and organic-acid biosynthesis). In CCT8089, shifts in acid profiles across phosphate sources are consistent with these mechanisms: with Ca_3_(PO_4_)_2_, higher malate and acetate production can both acidify the medium and chelate Ca²^+^, promoting calcium release and increasing soluble P; with FePO_4_, higher gluconic acid production aligns with its affinity for metal ions and capacity to complex Fe³^+^, thereby facilitating Fe-phosphate dissolution (Rawat et al., 2021).

On the other hand, the observed lactic acid production in the presence of AlPO_4_ suggests a specific aluminum chelation mechanism, which reduces Al toxicity and increases the bioavailable phosphorus fraction in the solution (Hirozawa et al., 2023). Additionally, the exudation of this acid may be associated with an aluminum tolerance mechanism like that observed in plants that secrete organic acids to neutralize the toxic effects of Al³^+^, while simultaneously solubilizing adsorbed phosphorus (Panhwar et al., 2015).

In addition to solubilizing and mineralizing phosphate, these *Bacillus* strains (CCT8089, B119 and B2084) also demonstrated the ability to produce IAA, an essential phytohormone for root growth. IAA stimulates root development, increasing the absorptive surface area and enhancing nutrient uptake, including phosphorus (de Sousa et al., 2021). This root growth-promoting ability was further confirmed in greenhouse experiments, where higher root biomass was observed in the CCT8089-inoculated treatment compared to the non-inoculated control.

Based on the field results, inoculation with strain CCT8089 and the BiomaPhos inoculant (B119 and B2084) led to increased yield in soybean and maize crops, with furrow application in maize showing powerful results. These inoculants allowed treatments with reduced phosphorus fertilization to achieve comparable yields to those obtained with complete phosphorus fertilization in the control. Furthermore, these treatments enhanced PUE, demonstrating the potential of these microorganisms to stimulate root growth, solubilize phosphorus, and mineralize organic P, making it more available to plants. These findings align with previous studies (Mosela et al., 2022; Song et al., 2022; de Oliveira-Paiva et al., 2024; Torres et al., 2024), highlighting the importance of *Bacillus* species as bioinputs that not only promote plant growth but also improve PUE. This reduces dependence on synthetic phosphate fertilizers and supports adopting more sustainable agricultural practices.

The stability index analysis based on Lin and Binns (1988) revealed that inoculation with *Bacillus*, particularly strain CCT8089, contributed to greater yield stability, ensuring a more uniform productive response across varying environmental conditions. These results highlight the potential of strain CCT8089 as a novel inoculant for maize and soybean crops, with viable application through both seed treatment and furrow application.

## 5 Conclusions

*Bacillus siamensis* CCT8089 exhibits a robust suite of plant growth–promoting traits, organic-acid secretion, phosphatase/phytase activity, siderophore and IAA production, EPS/biofilm formation, and tolerance to osmotic stress, and effectively colonizes soybean roots. In greenhouse assays, CCT8089 increased root biomass. Across replicated, multi-environment field trials, inoculation (seed or in-furrow) improved grain yield and phosphorus acquisition efficiency in maize and soybean relative to the non-inoculated control at 83.25 kg P_2_O₅ ha⁻¹. In maize, in-furrow application outperformed seed treatment, with best performance at 200 mL ha⁻¹. In soybean, application modes performed similarly, with seed treatment effective at 100 mL per 50 kg of seed. Notably, CCT8089 delivered greater yield stability across contrasting environments. Together, these findings position CCT8089 as a promising PSB inoculant for tropical agriculture, with potential to enhance P use efficiency and reduce reliance on mineral P inputs.

## Data Availability

The raw data supporting the conclusions of this article will be made available by the authors, without undue reservation.
