# Supplementary material for: Bacillus siamensis CCT8089: a novel phosphate-solubilizing bacterium enhancing maize and soybean growth
Source: Front Plant Sci. 2025 Oct 16;16:1671339. doi: 10.3389/fpls.2025.1671339 (PMC12571876; doi:10.3389/fpls.2025.1671339)
Supplement: Supplementary file 1 [file Table1.docx]

***Bacillus siamensis* CCT8089: a novel phosphate-solubilizing bacterium enhancing maize and soybean growth**

Mirela Mosela^1^, Galdino Andrade^1^, Alison Fernando Nogueira^2^, Lycio Shinji Watanabe^3^, Silas Mian^2^, Matheus Felipe de Lima Andreata^1^, Marcos Ventura Faria^4^, Liliane Scislowski^4^, Daniel Fernando Viana Fagundes^2^, Antony Wallace Marcos^2^, Henry Boguschi Cava^2^, Pablo Diego Silva Cabral^5^, Roger Wisniewski da Conceição^5^, Sérgio Vicente de Azevedo^6^, Liliam Silvia Candido^7^, Leandro Afonso^1^, Rafael Assis^8^ and Leandro Simões Azeredo Gonçalves^2*^

^1^Microbiology Department, Universidade Estadual de Londrina (UEL), Londrina, Paraná, 86057-970, Brazil

^2^Agronomy Department, Universidade Estadual de Londrina (UEL), Londrina, Paraná, 86057-970, Brazil

^3^Chemical Departament, Universidade Estadual de Londrina (UEL), Londrina, Paraná, 86057-970, Brazil

^4^Agronomy Department, Universidade Estadual do Centro Oeste (UNICENTRO), Guarapuava, Paraná, 85040-167, Brazil

^5^Agronomy Department, Instituto Federal Goiano (IFG), Rio Verde, Goiás, 75901-970, Brazil

^6^Biology Department, Instituto Federal de São Paulo (IFSP), Barretos, São Paulo, 14781-502, Brazil

^7^ Biology Department, Universidade Federal de Grande Dourados (UFGD), Dourados, Mato Grosso do Sul, 79804-970, Brazil

^8^Structural Biology Department, Universidade Estadual de Ponta Grossa (UEPG), Ponta Grossa, Paraná, 84030-900, Brazil

*Corresponding author: leandrosag@uel.br

**SUPPLEMENTARY TABLE**

**Supplementary Table S1.** Characterization of environments used in maize and soybean experiments.

| **Characteristics^1/^** | **Londrina - PR** | **Guarapuava - PR** | **Faxinal - PR** | **Dourados - MS** | **Barretos – SP** | **Rio Verde - GO** |
| --- | --- | --- | --- | --- | --- | --- |
| Geographical coordinates | 23^o^ 17`S; 51^o^ 10`W | 25^o^ 23`S; 51^o^ 29`W | 25^o^ 23`S; 51^o^ 29`W | 22^o^ 13`S; 54^o^ 59`W | 20^o^ 30`S; 48^o^ 33`W | 17^o^ 48`S; 50^o^ 54`W |
| Altitude (m) | 550 | 1026 | 895 | 409 | 565 | 745 |
| Climate^2/^ | Cfa | Cfb | Cfb | Am | Aw | Aw |
| Soil | Dystroferric Red Latosol | Dystroferric Bruno Latosol | Dystroferric Red Latosol | Dystroferric Red Latosol | Red-yellow Latosol | Red nitosolic latosol |
| pH(CaCl_2_) | 4.9 | 5.1 | 4.9 | 5.3 | 5.1 | 5.2 |
| H+Al (cmolc dm^3^) | 6.2 | 5.9 | 6.0 | 3.4 | 5.8 | 3.9 |
| K (cmolc dm^3^) | 0.6 | 2.0 | 0.6 | 0.4 | 0.6 | 0.3 |
| Ca (cmolc dm^3^) | 5.9 | 5.7 | 5.8 | 3.7 | 5.7 | 2.2 |
| Mg (cmolc dm^3^) | 2.4 | 2.0 | 2.2 | 1.5 | 2.0 | 0.6 |
| Al (cmolc dm^3^) | 0.1 | 0.0 | 0.0 | 0.0 | 0.01 | 0 |
| P (cmolc dm^3^) | 16.1 | 11.7 | 16.8 | 13.8 | 11.7 | 12.1 |
| Organic Matter (%) | 4.0 | 5.7 | 5.8 | 2.2 | 4.7 | 2.9 |

^1/^ Physical-chemical analyses were performed using soil layer samples from 0 to 20 cm.

^2/^ Köppen climate classification = Cfa, Humid subtropical climate; Cfb: Temperate oceanic climate; Am: tropical monsoon climate; and Aw: tropical savanna climate.
